# Sirt5 desuccinylates Cdc42 to mediate osteoclastogenesis and bone remodeling in mice

**DOI:** 10.1016/j.gendis.2023.04.033

**Published:** 2023-07-03

**Authors:** Yuang Zhang, Jing Wang, Jing Luan, Chuanju Liu, Yazhou Cui, Jinxiang Han

**Affiliations:** a Department of Orthopedics, The First Affiliated Hospital of Shandong First Medical University & Shandong Provincial Qianfoshan Hospital, Ji'nan, Shandong 250014, China; b Biomedical Sciences College & Shandong Medicinal Biotechnology Centre, Shandong First Medical University & Shandong Academy of Medical Sciences, Ji'nan, Shandong 250117, China; c NHC Key Laboratory of Biotechnology Drugs (Shandong Academy of Medical Sciences), Ji'nan, Shandong 250117, China; d Key Lab for Rare & Uncommon Diseases of Shandong Province, Ji'nan, Shandong 250117, China; e Department of Orthopaedic Surgery, New York University Grossman School of Medicine, New York, NY 10003, USA; f Department of Cell Biology, New York University Grossman School of Medicine, New York, NY 10016, USA

Post-translational modifications (PTMs) play a critical role in bone remodeling, with phosphorylation and acetylation being particularly well characterized. Recently, succinylation, a relatively uncommon PTM on lysine, has received considerable research attention for its influence on several physiological and pathological processes and conditions.^1^ Several substrates involved in mitochondrial pathways have been validated as substrates for the desuccinylase sirtuin 5 (Sirt5), a key regulator of succinylation.^2^ Bone is one of the most metabolically active organs, but the role of succinylation during bone remodeling is not well characterized.

In this study, we report that Sirt5-mediated desuccinylation is involved in bone remodeling. Mechanistically, we confirm that cell division cycle 42 (Cdc42), a critical regulator of osteoclast function, is a physiological substrate of Sirt5. We show that loss of Sirt5 stabilizes Cdc42 by succinylating lysine residue K153, which in turn protects Cdc42 from ubiquitination and subsequent degradation. Thus, our data reveal a mechanism by which Sirt5 mediates osteoclastogenesis by regulating Cdc42 succinylation and activity. It also suggests a potential intervention strategy for osteoporosis and other Cdc42-associated diseases.

First, we examined the effects of Sirt5 deficiency on bone remodeling in mice and showed that *Sirt5*^*−/−*^ mice had reduced trabecular bone compared to wild-type controls (Fig. 1A), a significant decrease in bone mineral density and trabecular thickness, and an increase in trabecular separation (Fig. S1A). Dynamic histomorphometric analysis using double labeling with tetracycline and calcein showed no difference in mineral apposition rate (Fig. S1B). Bone resorption marker fTRAP and serum calcium levels were significantly higher in *Sirt5*^*−/−*^ mice, whereas there were no significant differences in bone formation marker BALP levels (Fig. S1C). Histological staining showed that the number of osteoclasts was significantly increased in *Sirt5*^*−/−*^ mice (Fig. S1D), and *Sirt5* KO did not affect the number of osteoblasts (Fig. S1E). *Ex vivo* studies further showed that loss of Sirt5 could significantly enhance osteoclastogenesis (Fig. 1B), but did not affect osteogenesis (Fig. S2A). The expression of Sirt5 decreased during osteoclast differentiation of RAW264.7 preosteoclast cells and primary mouse bone marrow-derived macrophage cells (BMDMs) (Fig. S2B, C). The Sirt5-specific inhibitor MC3482 promoted osteoclast differentiation of RAW264.7 and BMDMs (Fig. S2D, E), whereas the Sirt5 activator resveratrol reversed this effect (Fig. S2F).

**Figure 1:**
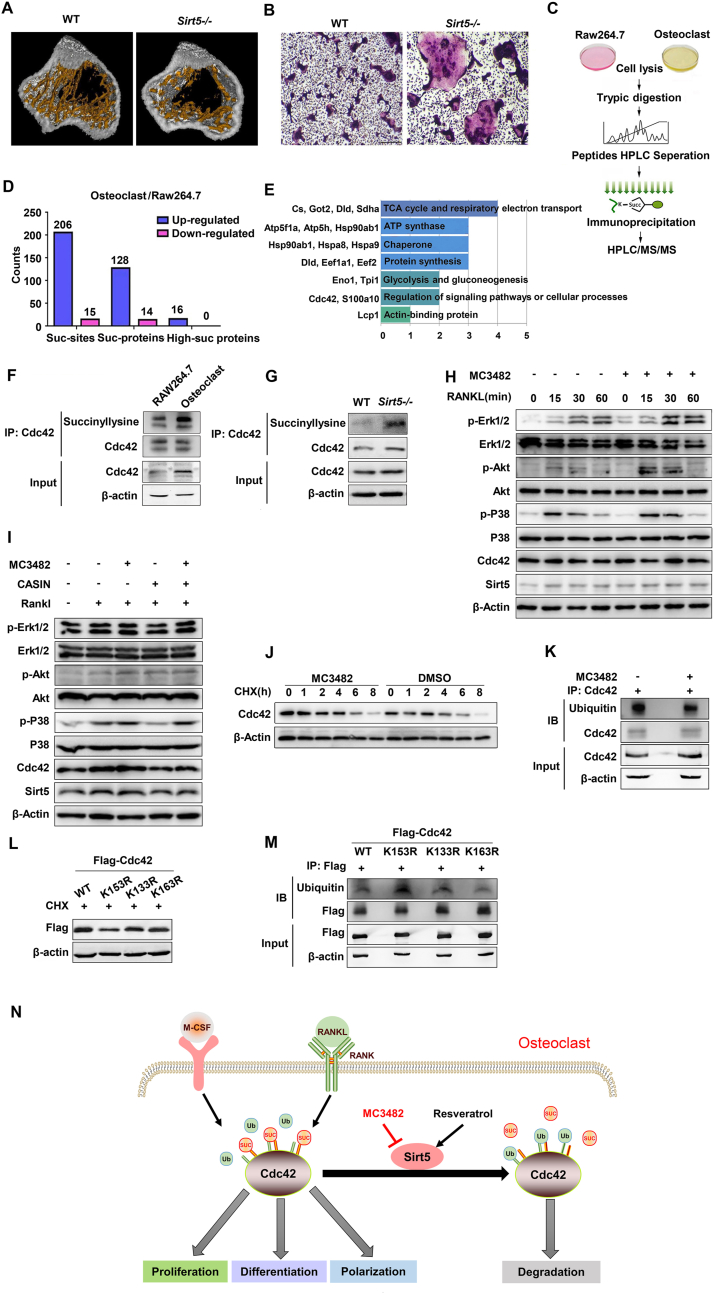
Sirt5 desuccinylates Cdc42 to mediate osteoclastogenesis and bone remodeling. **(A)** Representative images of tibial trabecular bone. **(B)** BMDMs isolated from *Sirt5*^*−/−*^ mice showed increased osteoclastogenesis as determined by Trap staining. **(C)** Experimental procedure for mass spectrometry-based lysine succinylome analysis. **(D)** Number of succinylated proteins and peptides and highly succinylated targets identified in RAW264.7 cells and osteoclasts. **(E)** Functional classification of highly succinylated targets. **(F)** CO-IP and Western blot assays validated higher succinylation status in differentiated osteoclasts. **(G)** Cdc42 was also highly succinylated in osteoclasts from *Sirt5*^*−/−*^ mice. **(H, I)** SIRT5 inhibition by MC3482 promoted RANKL-stimulated Cdc42-mediated p-Erk1/2, p-Akt, and p-38 activation, which can be attenuated by the Cdc42 inhibitor CASIN. **(J, K)** Sirt5 inhibitors can delay Cdc42 degradation in the CHX chase and increase ubiquitin modification levels. **(L, M)** Lys153 desuccinylation mutation showed a significant effect on Cdc42 degradation and ubiquitination. **(N)** A proposed model for explaining the effects of Sirt5 on Cdc42 modification and functions in osteoclasts.

We then investigated the precise mechanisms of Sirt5-mediated osteoclast differentiation. The levels of succinylation, but not malonylation and glutarylation, were found to increase dynamically during osteoclast differentiation of RAW264.7 cells (Fig. S3A). Lysine succinylome analysis identified a list of succinylated proteins during osteoclast differentiation of RAW264.7 cells (Fig. 1C and Table S1). The majority of these succinylated proteins and sites were identified in mature osteoclasts (Fig. 1D). As expected, the succinylated substrates were mainly cytoplasmic or mitochondrial proteins and enriched in different metabolic pathways. Among these identified targets, several known succinylated proteins including IDH2, PKM, S100A10, and SDHA have been reported in previous studies. Furthermore, based on the characteristics of the succinylated sites, we narrowed down this list to 16 highly succinylated targets that could contribute most to the effect of Sirt5, using the following criteria: (i) ≥3 succinylated sites; (ii) all succinylated sites show the same up- or down-trends in osteoclastogenesis; (iii) succinylated housekeeping proteins were eliminated. Finally, 16 highly succinylated proteins were selected (Fig. S3B). Most of the possible biological processes were focused on mitochondrial metabolism and ATP production (Fig. 1E), which are required for the energy-consuming processes of osteoclastogenesis and osteolysis. All highly succinylated proteins belong to up-regulated targets in osteoclastogenesis and no down-regulated ones were identified (Fig. S3B).

Most interestingly, among the highly succinylated targets, a well-known mediator of osteoclastogenesis, Cdc42, was found to be modified by succinylation at three lysine sites (Lys153, Lys133, Lys163) (Fig. S4A–C). Succinylation of Cdc42 has not been reported previously. Using a Co-IP assay, we further confirmed that Cdc42 succinylation levels were significantly increased in differentiated osteoclasts (Fig. 1F). Cdc42 was also more highly succinylated in osteoclasts isolated from *Sirt5* KO mice than from wild-type mice (Fig. 1G). Taken together, these results indicate that Cdc42 is succinylated during osteoclastogenesis.

Subsequently, Co-IP and Western blotting with Flag-Cdc42 and HA-Sirt5 ectopically expressed HEK293T cells showed that Cdc42 interacts with Sirt5 (Fig. S5A). Immunofluorescence staining revealed that endogenous Cdc42 colocalized with endogenous Sirt5 mainly in the cytoplasm of osteoclasts (Fig. S5B). We further showed that a Sirt5-specific inhibitor, MC3482, could enhance Cdc42-mediated activation of p-Erk1/2, p-Akt, and p-38 in RANKL-stimulated osteoclastogenesis (Fig. 1H), whereas a Cdc42 inhibitor, CASIN, could reverse this activation (Fig. 1I). *Sirt5* knockdown increased Cdc42 levels in RAW264.7 cells (Fig. S5C), whereas Sirt5 overexpression decreased Cdc42 levels in RAW264.7 cells, which was independent of RANKL stimulation (Fig. S5D). Primary osteoclasts from *Sirt5*^*−/−*^ mice also had increased Cdc42 expression compared to those from wild-type mice, and the Sirt5 activator resveratrol decreased Cdc42 levels in primary osteoclasts from wild-type mice, but had no effect in pre-osteoclasts from *Sirt5*^*−/−*^ mice (Fig. S5E). Furthermore, the reduced effects of resveratrol on Cdc42 could also be counteracted by the Sirt5 inhibitor MC3482 (Fig. S5F). Taken together, these data indicate that Cdc42 could be desuccinylated as a direct target of Sirt5 and suggest that Sirt5-mediated succinylation of Cdc42 affects its levels and corresponding activity in regulating RANKL-triggered osteoclastogenesis.

Finally, we found that the Sirt5 inhibitor MC3482 delayed the degradation and increased the protein stability of Cdc42 in the CHX chase (Fig. 1J). In addition, MC3482 treatment also decreased the ubiquitination of Cdc42 in RAW264.7 cells (Fig. 1K). K153 overlaps with two other sites (K133, K163) adjacent to known ubiquitination sites, suggesting a possible competition mechanism between succinylation and ubiquitination (Fig. S6A). Furthermore, K133 and K153 are highly conserved across species (Fig. S6B). We generated K to R mutants (mimicking desuccinylation) of Cdc42, including K133R, K153R, and K163R. As a result, the desuccinylation mimicking mutant K153R increased its ubiquitination level and promoted the degradation of Cdc42 (Fig. 1L, M). In contrast, K133R and K163R did not show any significant changes (Fig. 1L, M). Taken together, these results indicate that succinylation at the K153 residue protects Cdc42 from ubiquitination and degradation and that Sirt5 can abolish the protective effect by desuccinylation (Fig. 1N).

Given the central role of Cdc42 in bone remodeling,^3^ we propose that Cdc42 may be a key effector in determining the bone loss phenotype in the *Sirt5* null mice. In addition to Cdc42, we also found that several key enzymes involved in the tricarboxylic acid cycle and ATP production were also highly succinylated in osteoclasts, suggesting that there may be a significant Sirt5-mediated metabolic shift to accommodate energy-consuming osteoclastogenesis. Subsequently, changes in metabolites, such as ATP, would also regulate osteoclast survival and function.

Based on our findings, we propose that targeting Sirt5 to block Cdc42 activity may be an option for the treatment of osteoporosis. The Sirt5 activator resveratrol has been shown to have therapeutic potential in osteoporosis.^4^ More interestingly, Cdc42 inhibition has been shown to extend the lifespan of aged mice.^5^ Given the concurrent role of Sirt5, it is reasonable to speculate that the role of Sirt5-mediated Cdc42 desuccinylation may not be limited to osteoclasts, but may represent a common mechanism in the regulation of the aging process, where targeting Sirt5-Cdc42 may be a valuable intervention strategy for other age-related diseases.

## Conflict of interests

The authors declared no competing interests.

## Funding

The study was supported by the Academic Promotion Programme of Shandong First Medical University (China) (No. 2019LJ001), the 10.13039/501100001809National Natural Science Foundation of China (No. 81772300, 82101903), and The Innovation Project of Shandong Academy of Medical Sciences, Shandong, China.

## Figures and Tables

**Fig. S1:**
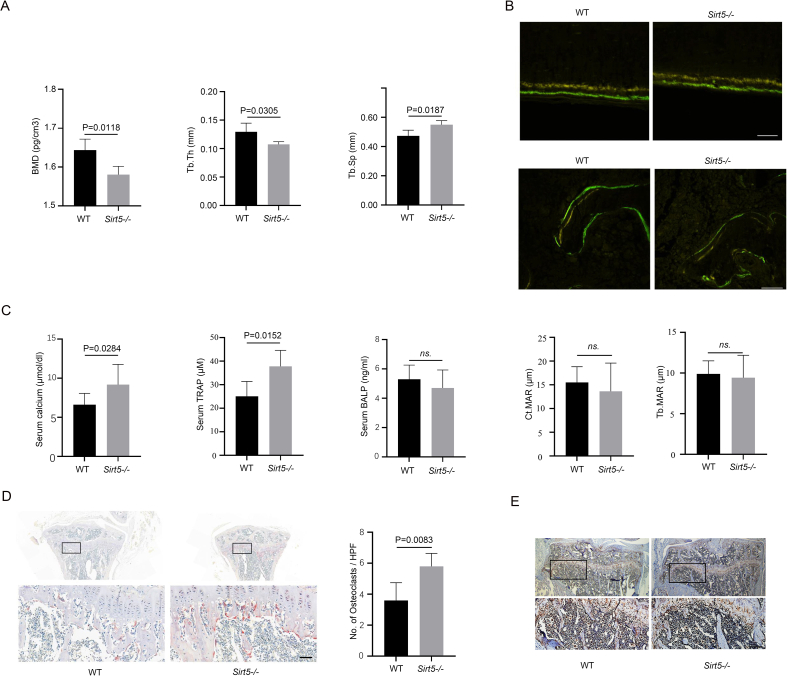
Bone remodeling of *Sirt5*^*−/−*^ mice and WT controls.

**Fig. S2:**
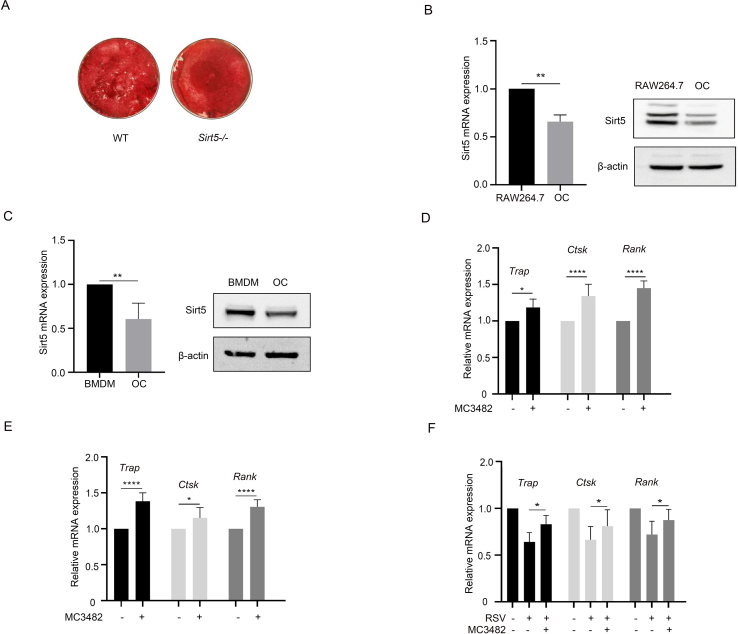
Sirt5 is associated with increased osteoclast differentiation.

**Fig. S3:**
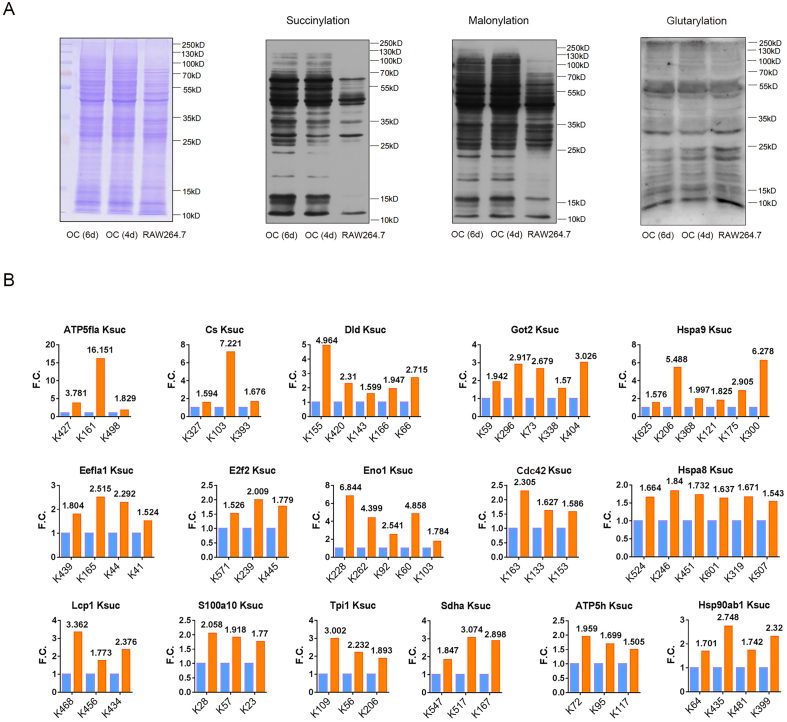
Lysine succinylome and SirtT5 expression during osteogenesis.

**Fig. S4:**
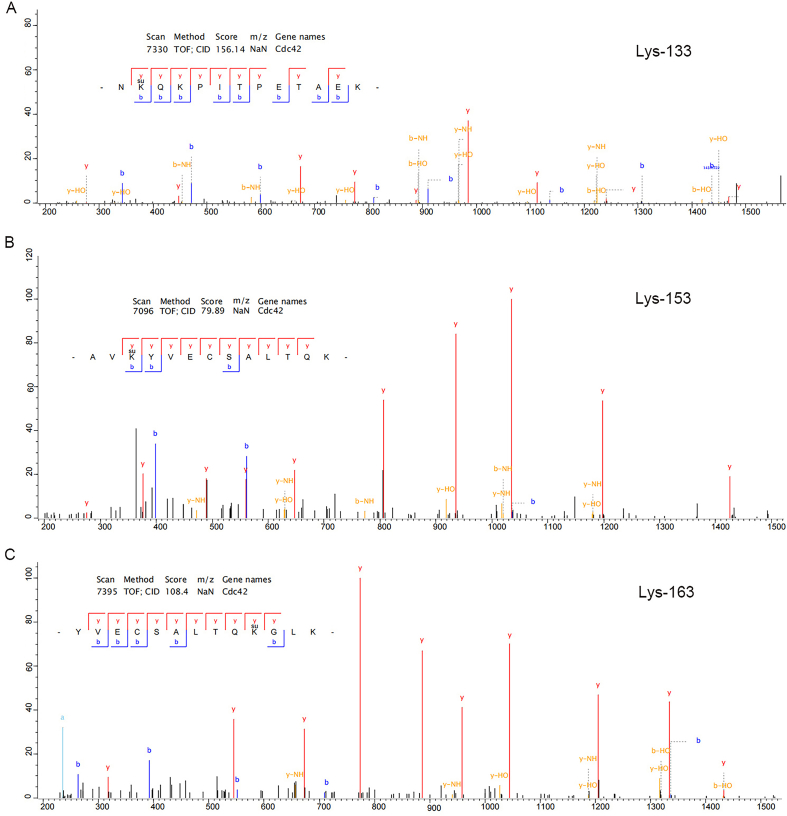
Cdc42 is a succinylated target of Sirt5.

**Fig. S5:**
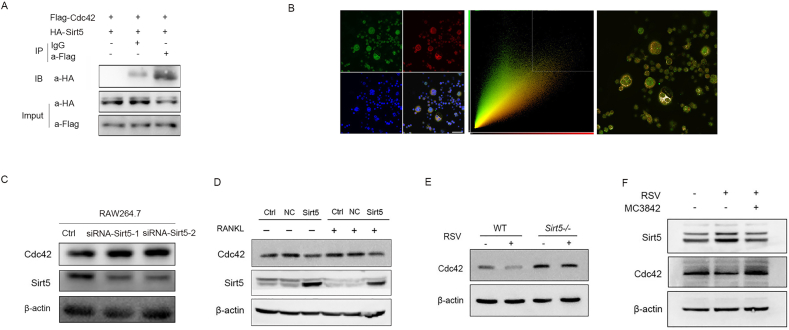
Sirt5 interacts with Cdc42 and regulates Cdc42 levels and signaling pathways.

**Fig. S6:**
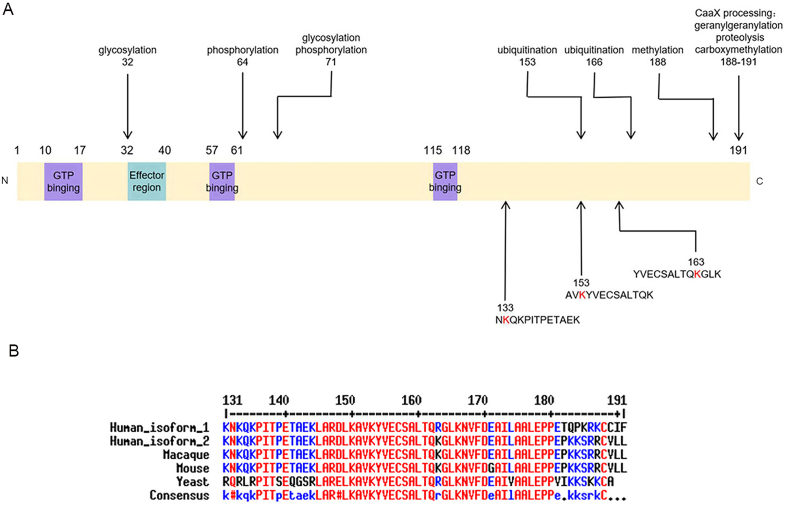
Identification of functional succinylated sites of Cdc42.

## Appendix A Supplementary data

Multimedia component 1 Table S1: A list of proteins that undergo succinylation modification after RAW264.7 osteoclastogenesis. Multimedia component 1
