# Supplementary material for: Sirt5 desuccinylates Cdc42 to mediate osteoclastogenesis and bone remodeling in mice
Source: Genes Dis. 2023 Jul 3;11(3):101002. doi: 10.1016/j.gendis.2023.04.033 (PMC10806281; doi:10.1016/j.gendis.2023.04.033)
Supplement: Multimedia component 1 — Table S1: A list of proteins that undergo succinylation modification after RAW264.7 osteoclastogenesis. [file mmc1.pdf]

A list of proteins undergoing succinylation modification after RAW264.7 osteoclastogenesis

Protein accession	Position	OC/Raw264.7 Ratio	Regulated Type	Amino acid	Protein description	Gene name	Localization probability
D3Z7P3	169	2.072	Up	K	"Glutaminase kidney isoforr	Gls	1
O08749	155	4.964	Up	K	"Dihydrolipoyl dehydrogena	Dld	1
O08749	420	2.31	Up	K	"Dihydrolipoyl dehydrogena	Dld	1
O08749	143	1.599	Up	K	"Dihydrolipoyl dehydrogena	Dld	1
O08749	166	1.947	Up	K	"Dihydrolipoyl dehydrogena	Dld	1
O08749	66	2.715	Up	K	"Dihydrolipoyl dehydrogena	Dld	1
O35381	28	2.272	Up	K	Acidic leucine-rich nuclear p	Anp32a	1
O35381	68	1.724	Up	K	Acidic leucine-rich nuclear p	Anp32a	1
P05064	322	1.607	Up	K	Fructose-bisphosphate aldol	Aldoa	1
P05064	42	1.607	Up	K	Fructose-bisphosphate aldol	Aldoa	1
P05202	59	1.942	Up	K	"Aspartate aminotransferase	Got2	1
P05202	296	2.917	Up	K	"Aspartate aminotransferase	Got2	1
P05202	73	2.679	Up	K	"Aspartate aminotransferase	Got2	1
P05202	338	1.57	Up	K	"Aspartate aminotransferase	Got2	1
P05202	404	3.026	Up	K	"Aspartate aminotransferase	Got2	1
P05213	336	1.984	Up	K	Tubulin alpha-1B chain OS=	Tuba1b	1
P07356	47	3.033	Up	K	Annexin A2 OS=Mus muscu	Anxa2	1
P08003	355	1.804	Up	K	Protein disulfide-isomerase .	Pdia4	1
P08113	537	2.138	Up	K	Endoplasmin OS=Mus musc	Hsp90b1	1
P08113	633	2.305	Up	K	Endoplasmin OS=Mus musc	Hsp90b1	1
P08207	28	2.058	Up	K	Protein S100-A10 OS=Mus	S100a10	0.999985
P08207	57	1.918	Up	K	Protein S100-A10 OS=Mus	S100a10	1
P08207	23	1.77	Up	K	Protein S100-A10 OS=Mus	S100a10	1
P08249	328	2.006	Up	K	"Malate dehydrogenase, mit	Mdh2	0.965796
P09103	116	0.516	Down	K	Protein disulfide-isomerase (	P4hb	1
P09405	431	2.667	Up	K	Nucleolin OS=Mus musculu	Ncl	1
P09411	6	2.554	Up	K	Phosphoglycerate kinase 1 (	Pgk1	1
P09411	11	1.703	Up	K	Phosphoglycerate kinase 1 (	Pgk1	1
P09411	267	2.067	Up	K	Phosphoglycerate kinase 1 (	Pgk1	1
P09671	130	2.322	Up	K	"Superoxide dismutase [Mn]	Sod2	1
P09671	122	1.569	Up	K	"Superoxide dismutase [Mn]	Sod2	1
P10126	439	1.804	Up	K	Elongation factor 1-alpha 1 (	Eef1a1	1
P10126	165	2.515	Up	K	Elongation factor 1-alpha 1 (	Eef1a1	1
P10126	44	2.292	Up	K	Elongation factor 1-alpha 1 (	Eef1a1	1
P10126	41	1.524	Up	K	Elongation factor 1-alpha 1 (	Eef1a1	1
P11499	64	1.701	Up	K	Heat shock protein HSP 90-	Hsp90ab1	1
P11499	435	2.748	Up	K	Heat shock protein HSP 90-	Hsp90ab1	1
P11499	481	1.742	Up	K	Heat shock protein HSP 90-	Hsp90ab1	1
P11499	399	2.32	Up	K	Heat shock protein HSP 90-	Hsp90ab1	1
P11983	400	0.499	Down	K	T-complex protein 1 subunit	Tcp1	1
P14211	151	1.613	Up	K	Calreticulin OS=Mus muscu	Calr	1
P15532	124	12.649	Up	K	Nucleoside diphosphate kina	Nme1	1
P16110	190	1.898	Up	K	Galectin-3 OS=Mus muscul	Lgals3	1
P16858	213	1.89	Up	K	Glyceraldehyde-3-phosphate	Gapdh	1
P16858	192	1.548	Up	K	Glyceraldehyde-3-phosphate	Gapdh	1
P17182	228	6.844	Up	K	Alpha-enolase OS=Mus mus	Eno1	1
P17182	262	4.399	Up	K	Alpha-enolase OS=Mus mus	Eno1	1
P17182	92	2.541	Up	K	Alpha-enolase OS=Mus mus	Eno1	1
P17182	60	4.858	Up	K	Alpha-enolase OS=Mus mus	Eno1	1
P17182	103	1.784	Up	K	Alpha-enolase OS=Mus mus	Eno1	1

P17742	31	2.412	Up	K	Peptidyl-prolyl cis-trans isor	Ppia	1
P17751	109	3.002	Up	K	Triosephosphate isomerase (	Tpi1	1
P17751	56	2.232	Up	K	Triosephosphate isomerase (	Tpi1	1
P17751	206	1.893	Up	K	Triosephosphate isomerase (	Tpi1	1
P17918	13	2.175	Up	K	Proliferating cell nuclear ant	Pcna	1
P18155	286	1.555	Up	K	"Bifunctional methylenetetra	Mthfd2	1
P18760	114	1.924	Up	K	Cofilin-1 OS=Mus musculus	Cfl1	1
P18760	92	2.256	Up	K	Cofilin-1 OS=Mus musculus	Cfl1	1
P19783	67	1.907	Up	K	"Cytochrome c oxidase subu	Cox4i1	1
P24452	28	0.609	Down	K	Macrophage-capping protein	Capg	0.999997
P26041	143	10.093	Up	K	Moesin OS=Mus musculus (	Msn	1
P26350	21	2.277	Up	K	Prothymosin alpha OS=Mus	Ptma	1
P26443	191	1.785	Up	K	"Glutamate dehydrogenase 1	Glud1	1
P27773	366	1.946	Up	K	Protein disulfide-isomerase .	Pdia3	1
P27773	494	1.703	Up	K	Protein disulfide-isomerase .	Pdia3	1
P35486	77	1.896	Up	K	"Pyruvate dehydrogenase E1	Pdha1	1
P35564	234	1.553	Up	K	Calnexin OS=Mus musculus	Canx	1
P35564	58	4.976	Up	K	Calnexin OS=Mus musculus	Canx	1
P35979	41	1.867	Up	K	60S ribosomal protein L12 (	Rpl12	1
P35979	54	3.641	Up	K	60S ribosomal protein L12 (	Rpl12	1
P38647	625	1.576	Up	K	"Stress-70 protein, mitochon	Hspa9	1
P38647	206	5.488	Up	K	"Stress-70 protein, mitochon	Hspa9	1
P38647	368	1.997	Up	K	"Stress-70 protein, mitochon	Hspa9	1
P38647	121	1.825	Up	K	"Stress-70 protein, mitochon	Hspa9	1
P38647	175	2.905	Up	K	"Stress-70 protein, mitochon	Hspa9	1
P38647	300	6.278	Up	K	"Stress-70 protein, mitochon	Hspa9	1
P40124	80	1.505	Up	K	Adenylyl cyclase-associated	Cap1	1
P40124	62	2.099	Up	K	Adenylyl cyclase-associated	Cap1	1
P40142	241	1.551	Up	K	Transketolase OS=Mus mus	Tkt	1
P42125	76	1.971	Up	K	"Enoyl-CoA delta isomerase	Eci1	1
P42932	37	2.36	Up	K	T-complex protein 1 subunit	Cct8	1
P47738	430	2.021	Up	K	"Aldehyde dehydrogenase, r	Aldh2	1
P49312	3	1.838	Up	K	Heterogeneous nuclear ribon	Hnrnpa1	1
P50543	50	1.719	Up	K	Protein S100-A11 OS=Mus	S100a11	1
P50544	279	2.657	Up	K	"Very long-chain specific ac	Acadvl	1
P51881	147	0.35	Down	K	ADP/ATP translocase 2 OS=	Slc25a5	1
P51881	33	1.525	Up	K	ADP/ATP translocase 2 OS=	Slc25a5	1
P51881	155	1.978	Up	K	ADP/ATP translocase 2 OS=	Slc25a5	1
P52480	115	2.13	Up	K	Pyruvate kinase PKM OS=M	Pkm	1
P52480	498	2.468	Up	K	Pyruvate kinase PKM OS=M	Pkm	1
P52480	66	2.786	Up	K	Pyruvate kinase PKM OS=M	Pkm	1
P52480	62	0.469	Down	K	Pyruvate kinase PKM OS=M	Pkm	1
P54071	155	1.56	Up	K	"Isocitrate dehydrogenase [N	Idh2	1
P54071	280	1.661	Up	K	"Isocitrate dehydrogenase [N	Idh2	1
P54071	282	1.625	Up	K	"Isocitrate dehydrogenase [N	Idh2	1
P54071	199	0.23	Down	K	"Isocitrate dehydrogenase [N	Idh2	1
P54071	48	0.366	Down	K	"Isocitrate dehydrogenase [N	Idh2	1
P56391	13	1.581	Up	K	Cytochrome c oxidase subun	Cox6b1	1
P58252	571	1.526	Up	K	Elongation factor 2 OS=Mus	Eef2	1
P58252	239	2.009	Up	K	Elongation factor 2 OS=Mus	Eef2	1
P58252	445	1.779	Up	K	Elongation factor 2 OS=Mus	Eef2	1
P60710	315	2.332	Up	K	"Actin, cytoplasmic 1 OS=M	Actb	1
P60710	50	3.168	Up	K	"Actin, cytoplasmic 1 OS=M	Actb	1
P60710	326	2.898	Up	K	"Actin, cytoplasmic 1 OS=M	Actb	1

P60766	163	2.305	Up	K	Cell division control protein	Cdc42	1
P60766	133	1.627	Up	K	Cell division control protein	Cdc42	0.999999
P60766	153	1.586	Up	K	Cell division control protein	Cdc42	1
P60843	174	1.616	Up	K	Eukaryotic initiation factor 4	Eif4a1	1
P61161	322	1.631	Up	K	Actin-related protein 2 OS=1	Actr2	1
P61979	405	2.596	Up	K	Heterogeneous nuclear ribor	Hnrnpk	1
P61982	69	1.577	Up	K	14-3-3 protein gamma OS=M	Ywhag	1
P61982	142	1.654	Up	K	14-3-3 protein gamma OS=M	Ywhag	1
P62259	69	0.189	Down	K	14-3-3 protein epsilon OS=M	Ywhae	1
P62830	113	1.554	Up	K	60S ribosomal protein L23 O	Rpl23	1
P62960	79	4.22	Up	K	Nuclease-sensitive element-l	Ybx1	1
P62983	48	2.459	Up	K	Ubiquitin-40S ribosomal pro	Rps27a	1
P63017	524	1.664	Up	K	Heat shock cognate 71 kDa	Hspa8	0.997731
P63017	246	1.84	Up	K	Heat shock cognate 71 kDa	Hspa8	1
P63017	451	1.732	Up	K	Heat shock cognate 71 kDa	Hspa8	1
P63017	601	1.637	Up	K	Heat shock cognate 71 kDa	Hspa8	1
P63017	319	1.671	Up	K	Heat shock cognate 71 kDa	Hspa8	1
P63017	507	1.543	Up	K	Heat shock cognate 71 kDa	Hspa8	1
P63038	359	0.566	Down	K	"60 kDa heat shock protein,	Hspd1	1
P63038	523	1.848	Up	K	"60 kDa heat shock protein,	Hspd1	1
P63038	396	1.926	Up	K	"60 kDa heat shock protein,	Hspd1	1
P63038	191	2.545	Up	K	"60 kDa heat shock protein,	Hspd1	1
P63101	9	2.501	Up	K	14-3-3 protein zeta/delta OS	Ywhaz	1
P63242	39	0.614	Down	K	Eukaryotic translation initiat	Eif5a	1
P67778	202	0.635	Down	K	Prohibitin OS=Mus musculu	Phb	1
P68254	9	1.528	Up	K	14-3-3 protein theta OS=Mu	Ywhaq	1
P80313	287	1.606	Up	K	T-complex protein 1 subunit	Cct7	1
P80313	55	1.521	Up	K	T-complex protein 1 subunit	Cct7	1
P80314	248	3.973	Up	K	T-complex protein 1 subunit	Cct2	1
P80317	127	1.656	Up	K	T-complex protein 1 subunit	Cct6a	1
P97372	156	1.739	Up	K	Proteasome activator compl	Psme2	1
P97807	77	2.915	Up	K	"Fumarate hydratase, mitoch	Fh	1
P99028	83	1.639	Up	K	"Cytochrome b-c1 complex	Uqcrh	1
Q01768	49	1.696	Up	K	Nucleoside diphosphate kina	Nme2	1
Q01853	658	1.625	Up	K	Transitional endoplasmic ret	Vcp	1
Q03265	427	3.781	Up	K	"ATP synthase subunit alpha	Atp5f1a	1
Q03265	161	16.151	Up	K	"ATP synthase subunit alpha	Atp5f1a	1
Q03265	498	1.829	Up	K	"ATP synthase subunit alpha	Atp5f1a	1
Q60864	530	2.042	Up	K	Stress-induced-phosphoprot	Stip1	1
Q61102	350	1.567	Up	K	"ATP-binding cassette sub-f	Abcb7	1
Q61233	468	3.362	Up	K	Plastin-2 OS=Mus musculus	Lcp1	1
Q61233	456	1.773	Up	K	Plastin-2 OS=Mus musculus	Lcp1	1
Q61233	434	2.376	Up	K	Plastin-2 OS=Mus musculus	Lcp1	1
Q61316	53	2.06	Up	K	Heat shock 70 kDa protein 4	Hspa4	1
Q61598	210	1.613	Up	K	Rab GDP dissociation inhib	Gdi2	1
Q61753	384	2.278	Up	K	D-3-phosphoglycerate dehyd	Phgdh	1
Q61937	248	1.992	Up	K	Nucleophosmin OS=Mus mu	Npm1	1
Q61937	150	1.705	Up	K	Nucleophosmin OS=Mus mu	Npm1	1
Q64433	8	1.635	Up	K	"10 kDa heat shock protein,	Hspe1	1
Q64433	28	0.536	Down	K	"10 kDa heat shock protein,	Hspe1	1
Q64433	40	2.633	Up	K	"10 kDa heat shock protein,	Hspe1	1
Q6IRU2	13	1.671	Up	K	Tropomyosin alpha-4 chain	Tpm4	1
Q8BH04	108	1.542	Up	K	"Phosphoenolpyruvate carbo	Pck2	1
Q8BH86	91	2.733	Up	K	"D-glutamate cyclase, mitoc	Dglucy	1

Q8BHN3	908	2.008	Up	K	Neutral alpha-glucosidase A	Ganab	1
Q8BIJ6	775	2.368	Up	K	"Isoleucine--tRNA ligase, m	Iars2	1
Q8BMD8	435	1.921	Up	K	Calcium-binding mitochond	Slc25a24	1
Q8BMS1	519	1.738	Up	K	"Trifunctional enzyme subu	Hadha	1
Q8C3X2	167	1.576	Up	K	"Coiled-coil domain-contain	Ccdc90b	1
Q8JZN5	242	2.359	Up	K	"Acyl-CoA dehydrogenase f	Acad9	1
Q8JZU0	339	3.481	Up	K	Nucleoside diphosphate-link	Nudt13	1
Q8K1R3	583	3.02	Up	K	"Polyribonucleotide nucleot	Pnpt1	1
Q8K2B3	547	1.847	Up	K	"Succinate dehydrogenase [u	Sdha	1
Q8K2B3	517	3.074	Up	K	"Succinate dehydrogenase [u	Sdha	1
Q8K2B3	167	2.898	Up	K	"Succinate dehydrogenase [u	Sdha	1
Q8K411	770	2.183	Up	K	"Presequence protease, mito	Pitrm1	1
Q8QZT1	260	1.537	Up	K	"Acetyl-CoA acetyltransfera	Acat1	1
Q8VDC0	719	2.524	Up	K	"Probable leucine--tRNA lig	Lars2	1
Q8VDD5	1173	0.658	Down	K	Myosin-9 OS=Mus musculu	Myh9	1
Q8VDD5	1445	2.087	Up	K	Myosin-9 OS=Mus musculu	Myh9	1
Q8VDD5	1392	1.53	Up	K	Myosin-9 OS=Mus musculu	Myh9	1
Q91V41	59	1.781	Up	K	Ras-related protein Rab-14 (	Rab14	1
Q91VD9	84	2.021	Up	K	"NADH-ubiquinone oxidore	Ndufs1	1
Q91VD9	467	1.798	Up	K	"NADH-ubiquinone oxidore	Ndufs1	1
Q91YQ5	580	1.501	Up	K	Dolichyl-diphosphooligosac	Rpn1	1
Q925I1	494	0.527	Down	K	ATPase family AAA domain	Atad3	1
Q93092	277	2.542	Up	K	Transaldolase OS=Mus mus	Taldo1	1
Q99020	237	1.575	Up	K	Heterogeneous nuclear ribon	Hnrnpab	1
Q99JB2	145	1.786	Up	K	"Stomatin-like protein 2, mi	Stoml2	1
Q99K85	117	1.632	Up	K	Phosphoserine aminotransfe	Psat1	1
Q99KE1	74	2.048	Up	K	"NAD-dependent malic enzy	Me2	1
Q99KI0	689	1.517	Up	K	"Aconitate hydratase, mitoch	Aco2	1
Q99KI0	739	1.804	Up	K	"Aconitate hydratase, mitoch	Aco2	0.999668
Q99KK9	90	1.532	Up	K	"Probable histidine--tRNA l	Hars2	1
Q99LC5	69	1.801	Up	K	"Electron transfer flavoprote	Etfa	1
Q99N84	154	1.898	Up	K	"28S ribosomal protein S18b	Mrps18b	1
Q9CQQ7	233	1.818	Up	K	"ATP synthase F(0) complex	Atp5f1	1
Q9CQV8	70	1.547	Up	K	14-3-3 protein beta/alpha OS	Ywhab	1
Q9CZL5	124	9.753	Up	K	Pterin-4-alpha-carbinolamin	Pcbd2	1
Q9CZN7	367	1.531	Up	K	"Serine hydroxymethyltrans	Shmt2	1
Q9CZN7	280	1.824	Up	K	"Serine hydroxymethyltrans	Shmt2	1
Q9CZU6	327	1.594	Up	K	"Citrate synthase, mitochond	Cs	1
Q9CZU6	103	7.221	Up	K	"Citrate synthase, mitochond	Cs	1
Q9CZU6	393	1.676	Up	K	"Citrate synthase, mitochond	Cs	1
Q9D0K2	455	4.538	Up	K	"Succinyl-CoA:3-ketoacid c	Oxct1	1
Q9D1A2	364	1.606	Up	K	Cytosolic non-specific dipep	Cndp2	1
Q9D1A2	301	1.715	Up	K	Cytosolic non-specific dipep	Cndp2	1
Q9D6R2	336	2.119	Up	K	"Isocitrate dehydrogenase [N	Idh3a	1
Q9DB20	199	1.665	Up	K	"ATP synthase subunit O, m	Atp5o	1
Q9DBJ1	251	2.02	Up	K	Phosphoglycerate mutase 1 (	Pgam1	1
Q9DBJ1	113	4.584	Up	K	Phosphoglycerate mutase 1 (	Pgam1	1
Q9DC69	157	2.404	Up	K	"NADH dehydrogenase [ubi	Ndufa9	1
Q9DC69	175	1.938	Up	K	"NADH dehydrogenase [ubi	Ndufa9	1
Q9DCX2	72	1.959	Up	K	"ATP synthase subunit d, mi	Atp5h	1
Q9DCX2	95	1.699	Up	K	"ATP synthase subunit d, mi	Atp5h	1
Q9DCX2	117	1.505	Up	K	"ATP synthase subunit d, mi	Atp5h	1
Q9EP89	270	0.581	Down	K	"Serine beta-lactamase-like	Lactb	1
Q9JLJ2	310	1.847	Up	K	4-trimethylaminobutyraldeh	Aldh9a1	1

Q9JLZ3	179	1.543	Up	K	"Methylglutaconyl-CoA hyd	Auh	1
Q9QUM9	45	1.555	Up	K	Proteasome subunit alpha ty	Psma6	1
Q9WTP7	20	3.546	Up	K	"GTP:AMP phosphotransfer	Ak3	1
Q9WUM5	90	3.01	Up	K	"Succinate--CoA ligase [AD	Suclg1	1
Q9WVA4	78	2.483	Up	K	Transgelin-2 OS=Mus musc	Tagln2	1
Q9Z0J0	105	1.637	Up	K	NPC intracellular cholestero	Npc2	1
Q9Z1J3	159	1.722	Up	K	"Cysteine desulfurase, mitoc	Nfs1	1
Q9Z1N5	334	1.827	Up	K	Spliceosome RNA helicase l	Ddx39b	1
Q9Z2I8	48	2.082	Up	K	"Succinate--CoA ligase [GD	Suclg2	1

PEP	Score	Modified sequence	Charge	Mass error [ppm]	MS/MS Count	Subcellular localization
3.09456E-12	162.49	FITALK(1)STGLR	2	2.4087	2	mitochondria
0.00332853	69.276	VVHVNGFGK(1)ITGK	2	1.9527	2	mitochondria
0.00104955	64.064	IGK(1)FPFAANSR	2	2.5832	2	mitochondria
2.4566E-21	207.6	ALTGGIAHLFK(1)QNK	2	4.0391	2	mitochondria
8.45026E-44	278.33	NQVTATK(1)ADGSTQVIDTK	2	4.8172	4	mitochondria
0.00716278	54.27	SAQLGFK(1)TVCIEK	2	3.5573	2	mitochondria
0.000447056	79.889	ELVLDNCK(1)SIEGK	2	4.5693	2	cytoplasm
0.00305365	75.243	K(1)LELSENR	2	0.57182	2	cytoplasm
1.33027E-06	151.66	ENLK(1)AAQEEYIK	2	5.558	4	cytoplasm
4.10073E-31	196.44	GILAADESTGSIAK(1)R	2	4.0358	2	cytoplasm
0.00183535	58.426	K(1)MNLGVGAYR	2	1.4801	2	mitochondria
4.90168E-08	117.21	VGAFTVVCK(1)DAEEAK	2	4.454	4	mitochondria
0.000122792	105.94	DDNGK(1)PYVLPSVR	2	3.7917	4	mitochondria
0.000519392	133.68	K(1)QWLQEVK	2	1.5522	2	mitochondria
1.75573E-08	139.02	EFSVYMTK(1)DGR	2	2.8367	4	mitochondria
0.000111211	88.24	DVNAAIATIK(1)TK	2	2.32	2	cytoskeleton
0.000888643	83.775	DALNIETAVK(1)TK	2	3.2658	2	cytoplasm
6.45572E-05	144.27	LVLTHPEK(1)FQSK	2	5.2083	2	extracellular
0.000112826	81.799	QDK(1)IYFMAGSSR	2	3.5252	2	endoplasmic reticulum
3.03895E-05	112.93	IEK(1)AVVSQR	2	-0.25506	4	endoplasmic reticulum
8.98303E-05	85.813	FAGDKDHLTK(1)EDLR	3	1.1085	2	cytoplasm
0.00697746	49.96	IMK(1)DLDQCR	2	1.8766	3	cytoplasm
0.00926352	72.365	FAGDK(1)DHLTK	2	1.6388	2	cytoplasm
0.00589638	60.547	ASIK(0.966)K(0.034)GEDFVK	2	2.9159	2	mitochondria
2.70642E-07	119.52	NGDTASPK(1)EYTAGR	2	3.7395	3	mitochondria
0.00326777	65.933	SK(1)GIAYIEFK	2	3.4669	2	nucleus
0.000228474	96.957	SLSNK(1)LTLDK	2	0.49199	2	cytoplasm
0.00504931	76.572	LTLDK(1)LDVK	2	1.606	2	cytoplasm
0.0135759	54.436	IVK(1)DLMSK	2	0.31761	3	cytoplasm
0.0158899	51.864	DFGSFEK(1)FK	2	2.4288	2	mitochondria
0.00284805	84.368	GELLEAIK(1)R	2	1.3262	2	mitochondria
3.47816E-10	165.09	QTVAVGVIK(1)AVDK	2	3.2939	4	cytoplasm
2.24571E-13	171.34	MDSTEPPYSQK(1)R	2	3.8672	4	cytoplasm
0.000159874	143.39	FEK(1)EAAEMGK	2	2.4261	6	cytoplasm
0.0263761	60.589	TIEK(1)FEK	2	-0.17033	2	cytoplasm
1.4519E-10	131.54	YESLTDPSK(1)LDSGK	2	4.9644	2	cytoplasm
0.00346176	60.892	FYEAFSK(1)NLK	2	2.6729	2	cytoplasm
6.32073E-06	108.5	ETQK(1)SIYYITGESK	2	5.0676	2	cytoplasm
0.000627781	98.009	EMLQQSK(1)ILK	2	1.7436	4	cytoplasm
0.000654829	67.881	SLHDALCVVK(1)R	2	4.0259	2	cytoplasm
0.000717934	101.41	VHVIFNYK(1)GK	2	1.626	3	endoplasmic reticulum
0.0031395	78.864	NIIHGSDSVK(1)SAEK	3	1.9228	2	cytoplasm
4.9164E-22	219.93	VIVCNTK(1)QDNNWGK	2	4.7543	2	cytoplasm
5.23315E-09	118.26	GAAQNIIPASTGAAK(1)AVGK	2	4.8801	2	cytoplasm
0.000383278	107.08	TVDGPSGK(1)LWR	2	1.5407	2	cytoplasm
0.000461694	108.93	EALELLK(1)TAIAK	2	1.6708	2	cytoplasm
0.000243516	115.04	YDLDFK(1)SPDDPSR	2	5.2126	2	cytoplasm
1.45872E-11	139.61	IDK(1)LMIEMDGTENK	2	5.5099	4	cytoplasm
6.43036E-08	138	FMGK(1)GVSQAVEHINK	2	4.0152	7	cytoplasm
9.56851E-08	172.1	LMIEMDGTENK(1)SK	2	3.8705	7	cytoplasm

0.00287409	99.305	VPK(1)TAENFR	2	0.81263	2	cytoplasm
8.46952E-05	96.946	LDPK(1)IAVAAQNCYK	2	5.6644	2	cytoplasm
0.00201229	82.716	K(1)FFVGGNWK	2	1.6083	2	cytoplasm
0.00315699	69.276	VIADNVK(1)DWSK	2	2.455	2	cytoplasm
0.00418052	60.589	LIQGSILK(1)K	2	0.062346	2	cytoplasm
0.00202161	85.716	VQDPVTAK(1)PK	2	1.4506	2	mitochondria
0.00120932	85.716	SK(1)MIYASSK	2	2.061	3	mitochondria
4.7325E-07	103.76	YALYDATYETK(1)ESK	2	4.6982	2	mitochondria
0.000820164	105.36	EK(1)ADWSSLSR	2	2.4598	2	mitochondria
0.00128178	117.3	VEK(1)LKPVPIAR	2	1.4572	2	cytoplasm,nucleus
1.53472E-05	124.06	EVHK(1)SGYLAGDK	2	2.191	2	cytoplasm
2.32819E-05	94.66	K(1)EVVEEAENGR	2	3.3759	2	cytoplasm
0.00100701	86.243	INPK(1)NYTDNELEK	2	4.1416	2	mitochondria
3.07643E-08	119.52	YLK(1)SEPIPESNEGPVK	2	4.591	2	endoplasmic reticulum
4.40843E-06	150.44	EATNPPIIQEEK(1)PK	2	3.005	2	endoplasmic reticulum
0.0032595	79.311	RMELK(1)ADQLYK	2	3.7463	3	mitochondria
0.0327955	53.679	TYFTDK(1)K	2	-0.67938	2	endoplasmic reticulum
0.00327255	54.27	SDASTPPSPK(1)VTYK	2	3.9678	2	endoplasmic reticulum
0.016647	79.059	K(1)VGDDIAK	2	-0.55965	2	cytoplasm
0.00544502	62.88	ATGDWK(1)GLR	2	2.1242	2	cytoplasm
7.94984E-08	160.62	ALLAGK(1)DSETGENIR	2	4.0513	2	mitochondria
4.9133E-32	246.68	QATK(1)DAGQISGLNVLR	2	4.3005	2	mitochondria
5.08354E-07	115.79	TIAPCQK(1)AMQDAEVSK	2	5.7236	5	mitochondria
4.90807E-06	104.04	QAVTNPNNTFYATK(1)R	2	4.0098	2	mitochondria
9.63602E-11	138.71	MK(1)ETAENYLGHTAK	2	4.8143	4	mitochondria
3.8152E-23	236.7	ETGVDLTK(1)DNMALQR	2	5.9619	6	mitochondria
6.26099E-05	88.24	HAEMVHTGLK(1)LER	3	0.74987	4	cytoplasm
0.000404801	103.87	MSK(1)EIGGDVQK	2	1.554	4	cytoplasm
0.000775224	71.896	HQPTAIIAK(1)TFK	2	2.7341	2	cytoplasm
0.00621548	93.561	LENDK(1)SIR	2	-1.4079	2	mitochondria
9.01587E-05	127.03	NIQACK(1)ELAQTTR	2	4.4395	2	cytoplasm
4.43394E-05	158.57	FK(1)TIEEVVGR	2	2.3569	2	mitochondria
8.23358E-07	130.71	SK(1)SESPKEPEQLR	2	2.7757	2	nucleus
0.0026092	90.761	NQK(1)DPGVLDR	2	0.13866	2	cytoplasm
0.00264282	64.064	EK(1)ITAFVVER	2	-0.30257	2	mitochondria
6.41256E-05	125.17	LAADVGK(1)AGAER	2	2.7313	3	cytoplasm
4.15426E-06	130.79	VK(1)LLLQVQHASK	2	3.7179	4	cytoplasm
0.000149197	116.13	EFK(1)GLGDCLVK	2	2.8895	2	cytoplasm
1.00967E-05	134.2	PVAVALDTK(1)GPEIR	2	3.9446	2	cytoplasm
0.00015399	117.3	VNLAMDVGK(1)AR	2	2.0234	5	cytoplasm
1.03667E-05	108.3	EMIK(1)SGMNVAR	2	2.1587	9	cytoplasm
0.0042284	63.397	SVEMLK(1)EMIK	2	1.8434	4	cytoplasm
0.000942531	98.009	EPIICK(1)NIPR	2	3.1284	2	mitochondria
0.0109768	76.833	TDFDK(1)NK	2	1.3654	2	mitochondria
0.00166651	90.308	NK(1)IWYEHR	2	2.624	4	mitochondria
0.00150062	57.888	LVFTPK(1)DGSSAK	2	2.4764	3	mitochondria
0.00135097	83.474	VEK(1)PVVEMDGDEMTR	2	4.2701	2	mitochondria
0.000722475	94.804	NYK(1)TAPFDSR	2	1.3113	2	extracellular
8.63303E-05	136.94	DLEEDHACIPIK(1)K	2	4.3403	2	cytoplasm
2.54225E-05	93.162	FAAK(1)GEGQLSAAER	2	3.1909	2	cytoplasm
0.000602658	121.31	EDLYLK(1)PIQR	2	4.04	3	cytoplasm
8.771E-16	187.51	MQK(1)EITALAPSTMK	2	4.6636	7	cytoskeleton
2.53503E-13	138.33	HQGVMVGMGQK(1)DSYVGD	3	4.8758	5	cytoskeleton
3.53174E-07	138.23	EITALAPSTMK(1)IK	2	3.1823	6	cytoskeleton

0.000231535	108.4	YVECSALTQK(1)GLK	2	3.8905	2	cytoplasm
9.63755E-07	156.14	NK(1)QKPITPETAEK	2	5.1235	2	cytoplasm
0.000447056	79.889	AVK(1)YVECSALTQK	2	3.0927	2	cytoplasm
0.00729122	77.072	YLSPK(1)YIK	2	0.26924	2	nucleus
0.0031929	50.834	ELK(1)QLYLER	2	2.4766	2	cytoplasm
9.70017E-05	85.622	DLAGSIIGK(1)GGQR	2	2.6393	2	nucleus
9.38131E-52	264.98	VISSIEQK(1)TSADGNEK	2	4.3121	2	cytoplasm
0.00078725	74.424	YLAEVATGEK(1)R	2	2.5432	2	cytoplasm
1.57199E-06	159.93	IISSIEQK(1)EENK	2	1.438	2	cytoplasm
0.000111921	103.76	GEMK(1)GSAITGPVAK	2	2.9283	2	cytoplasm
4.30278E-11	132.93	NDTK(1)EDVFVHQTAIK	2	6.0017	2	nucleus
2.74146E-06	126.29	LIFAGK(1)QLEDGR	2	3.1962	2	extracellular
1.18067E-10	146.58	MVQEAEK(0.998)YK(0.002)AE	2	3.5661	4	cytoplasm
9.07751E-05	90.142	MVNHFIAEFK(1)R	2	0.58878	2	cytoplasm
0.020159	57.032	AMTK(1)DNNLLGK	2	1.6018	2	cytoplasm
4.11015E-06	136.06	ELEK(1)VCNPIITK	2	3.0614	2	cytoplasm
0.000807414	81.099	GTLDPVEK(1)ALR	2	2.6699	2	cytoplasm
0.00676429	74.486	ITITNDK(1)GR	2	-6.0368	2	cytoplasm
0.00861295	74.486	DDAMLLK(1)GK	2	0.52774	3	mitochondria
0.0113571	56.378	GIIDPTK(1)VVR	2	1.694	2	mitochondria
0.00377552	72.922	LAK(1)LSDGVAVLK	2	3.0463	2	mitochondria
0.00021488	96.957	DIGNIISDAMK(1)K	2	4.1503	2	mitochondria
2.84804E-10	171.34	MDKNELVQK(1)AK	2	3.4305	4	cytoplasm
0.00364963	95.138	GRPCK(1)IVEMSTSK	2	3.7307	3	cytoplasm
4.27914E-06	150.53	FVVEK(1)AEQQK	2	0.35073	2	cytoplasm
0.0106436	56.014	TELIQK(1)AK	2	1.699	2	cytoplasm
0.000887996	81.48	IHQSGAK(1)VILSK	2	3.2597	2	cytoplasm
0.00151755	61.577	GK(1)ATISNDGATILK	2	3.1885	2	cytoplasm
0.000130278	86.243	ILIANTGMDTDK(1)IK	2	4.2858	2	cytoplasm
0.00345568	69.276	IITEGFEAAK(1)EK	2	4.2738	2	cytoplasm
0.00232768	94.791	TK(1)VEAFQTTISK	2	3.1734	2	cytoplasm
7.94272E-11	176.45	STMNFK(1)IGGATER	2	3.3248	4	mitochondria
0.000936323	88.181	DHCVAHK(1)LFK	2	3.1679	3	extracellular
0.000273052	78.157	ASEEHLK(1)QHYIDLK	3	2.3267	2	cytoplasm
0.0129322	58.286	VAILK(1)ANLR	2	0.52883	2	cytoplasm,nucleus
0.000399667	99.855	AMK(1)QVAGTMK	2	1.5529	6	mitochondria
4.69441E-14	148.57	VVDALGNAIDGK(1)GPIGSK	2	4.8692	2	mitochondria
0.00191206	72.365	GYLDK(1)LEPSK	2	1.6596	2	mitochondria
0.00296974	84.368	NPVIAQK(1)IQK	2	2.7623	2	cytoplasm
6.04737E-06	140.05	YFNNEK(1)YEAQR	2	4.9344	2	plasma membrane
0.000037478	96.313	KLENCNYAVDLGK(1)NQAK	3	2.2	3	cytoplasm
5.67239E-10	169.57	K(1)LENCNYAVDLGK	2	3.871	2	cytoplasm
0.00446763	58.426	IK(1)VPVDWNR	2	2.4458	2	cytoplasm
0.000184246	99.589	SIGAAAK(1)SQVISNAK	2	4.062	2	cytoplasm
0.00123413	76.605	IK(1)LYSESLAR	2	1.2916	2	endoplasmic reticulum
7.27043E-05	96.946	EASK(1)QADVNLVNAK	2	3.3996	2	cytoplasm
0.0037355	74.269	AK(1)MQASIEK	2	0.42108	3	nucleus
0.0124409	69.915	SAPGGGNK(1)VPQK	2	1.5669	2	nucleus
0.00942783	98.009	K(1)FLPLFDR	2	1.4947	2	mitochondria
0.00144894	65.12	SAAETVTK(1)GGIMLPEK	2	4.3252	2	mitochondria
2.80669E-33	191.49	SQGK(1)VLQATVVAVGSGGK	2	3.849	2	mitochondria
1.107E-22	203.93	K(1)IQALQQQADDAEDR	2	2.3946	2	cytoplasm
4.89251E-10	156.14	VESK(1)TVIVTPSQR	2	3.6814	2	mitochondria
0.00296974	60.589	TICPQLQK(1)YK	2	0.13872	2	nucleus

6.00219E-11	145.08	PAAVVLQTK(1)GSPESR	2	3.5576	2	plasma membrane
0.00002639	136.94	ITDSYK(1)QYDFGK	2	4.435	2	mitochondria
0.000493874	110.88	IVSK(1)EGVSGLYR	2	1.7685	2	mitochondria
5.81155E-23	183	TSK(1)DTTASAVAVGLR	2	4.0207	2	mitochondria
0.00308543	94.804	ADNK(1)LDINLER	2	2.9641	2	mitochondria
0.00266259	66.664	TEVVDSDGSK(1)TDK	2	4.8702	2	mitochondria
0.00494703	72.492	LAIAHHLIK(1)K	2	1.9027	2	nucleus
0.000152885	138.26	K(1)EILQIMNK	2	2.0359	4	mitochondria
0.000817813	90.306	ISQLYGDLK(1)HLK	2	4.6038	2	mitochondria
0.00378442	57.293	LNMQK(1)SMQNHAAVFR	3	2.2693	3	mitochondria
0.00166571	98.009	TEDGK(1)IYQR	2	6.2038	2	mitochondria
0.000491811	120.33	K(1)YLLNCDNMR	2	4.2851	7	mitochondria
0.0139261	62.88	VDFSK(1)VPK	2	0.11628	3	mitochondria
0.000387577	83.214	TSGTVPQPQLLNSK(1)EK	2	5.3165	2	mitochondria
0.0120088	65.156	EQEVSILK(1)K	2	1.2503	2	nucleus
0.000921814	96.946	K(1)FDQLLAEEK	2	2.4362	2	nucleus
0.000113675	116.13	LQK(1)DLEGLSQR	2	2.2823	2	nucleus
0.00399378	65.404	IIEVSGQK(1)IK	2	0.36818	2	cytoplasm
0.00131454	74.424	MCLVEIEK(1)APK	2	4.2148	3	mitochondria
6.22058E-05	108.4	HSFCEVLK(1)DAK	2	4.9846	2	mitochondria
0.0060819	67.507	K(1)DTYLENEK	2	2.9324	3	endoplasmic reticulum
0.00422585	46.447	YVLK(1)PATEGK	2	0.26046	2	cytoplasm
7.4849E-08	122.2	LAPALSVK(1)AAQTSDSEK	2	5.4978	2	cytoplasm
2.52773E-08	101.52	VAQPK(1)EVYQQQQYGSGGR	2	4.0877	2	nucleus
0.00900827	78.178	LSLDK(1)VFR	2	0.43354	2	mitochondria
0.00865706	64.064	K(1)FGTVNIVHPK	2	2.8693	2	cytoplasm,nucleus
0.00378313	83.623	K(1)MTSPLEK	2	1.96	3	mitochondria
0.0130773	69.905	AIITK(1)SFAR	2	1.7478	2	mitochondria
0.00165423	101.45	DFAPGKPLK(1)CVIK	2	3.2544	2	mitochondria
7.08021E-06	110.91	HGAK(1)GLDTPAFELK	2	4.2334	2	mitochondria
0.0028137	79.889	VVQDLCK(1)VAGVAK	2	3.1679	2	mitochondria
0.00308056	56.378	LTQAIQK(1)AR	2	0.83467	2	mitochondria
1.05497E-06	136.94	SISVQQEK(1)ETIAK	2	4.5274	2	mitochondria
0.000566572	90.263	VISSIEQK(1)TER	2	2.64	2	cytoplasm
0.000232672	90.142	RDVK(1)LAQFIEK	2	3.9899	3	extracellular
6.04978E-05	140.05	NAQAMADALLK(1)R	2	2.7536	4	mitochondria
2.12221E-07	138.71	YADVVTTTTHK(1)TLR	2	3.975	4	mitochondria
1.44971E-05	129.69	EVGKDVSDEK(1)LR	2	1.8693	4	mitochondria
0.00392893	83.358	GYSIPECQK(1)MLPK	2	3.4493	3	mitochondria
0.000380711	106.35	IVPNILLEQGK(1)AK	2	4.0636	2	mitochondria
1.77951E-06	157.77	IMEK(1)CTLPLTGK	2	3.4324	6	mitochondria
0.00173802	78.816	K(1)FAELQSPNK	2	1.9592	2	cytoplasm
6.07371E-10	176.45	DVGAETLLHSCK(1)K	2	3.3723	2	cytoplasm
8.87404E-15	157.77	IEAACFATIK(1)DGK	2	5.193	2	cytoplasm
0.00507209	69.716	YVDMSAK(1)SK	2	1.2547	3	mitochondria
0.000109095	109.88	AMEAVAAQGK(1)VK	2	1.0562	5	cytoplasm
0.00472302	76.572	HGEAQVK(1)IWR	2	2.7358	2	cytoplasm
0.000557967	73.655	AIAQASK(1)EAGVER	2	1.843	2	mitochondria
1.69163E-05	91.939	FIHVSHLNASMK(1)SSSK	3	3.3127	4	mitochondria
7.0415E-06	126.82	PGLVDDFEK(1)K	2	2.6198	6	cytoplasm
4.21698E-22	233	YTALVDQEEK(1)EDVK	2	4.4035	2	cytoplasm
2.17338E-07	139.02	IQEYEK(1)QLEK	2	2.1215	2	cytoplasm
0.000345901	107.08	AK(1)VEQDSEAR	2	0.87617	2	mitochondria
0.000102543	87.942	FINEVVK(1)QTQK	2	1.9102	2	cytoplasm

9.26918E-11	151.52	VAASSAK(1)MGLVETK	2	3.6079	4	mitochondria
0.00109886	87.942	GK(1)DCAVIVTQK	2	1.9927	2	cytoplasm
2.92543E-05	76.563	AVIMGAPGSGK(1)GTVSSR	2	4.6506	4	cytoplasm
0.0223709	56.202	LVGGTTPGK(1)GGQK	2	1.6833	2	mitochondria
1.29422E-11	150.44	LINSLYPEGQAPVK(1)K	2	3.7395	2	cytoplasm
0.00446763	65.404	DK(1)VYSYLNK	2	2.7025	2	extracellular
1.26196E-07	72.031	HLVTTQTEHK(1)CVLDSCR	3	4.8608	2	mitochondria
0.00136402	65.156	YQQFK(1)DFQR	2	1.4955	2	cytoplasm,nucleus
0.00043415	113.7	K(1)LMSEHGVR	2	1.5066	4	peroxisome
