# Supplementary material for: Sirt5 desuccinylates Cdc42 to mediate osteoclastogenesis and bone remodeling in mice
Source: Genes Dis. 2023 Jul 3;11(3):101002. doi: 10.1016/j.gendis.2023.04.033 (PMC10806281; doi:10.1016/j.gendis.2023.04.033)
Supplement: Multimedia component 2 [file mmc2.docx]

**Figure illustrations**

**Figure S1** Bone remodelling of *Sirt5-/-* mice and WT controls. **(A)** Quantification of trabecular BMD, Tb.Th, and Tb.Sp. **(B)** Representative images of the trabecular sections by double fluorescence labelling (scale bars, 10 μm). Quantification of Ct.MAR and Tb.MAR. **(C)** Quantification of serum bone remodelling markers. **(D)** TRAP staining of osteoclasts in trabecular bone section and quantification of osteoclast number. **(E)** Representative images of osteoblasts by osteopontin immunostaining.

**Figure S2** Sirt5 is associated with increased osteoclast differentiation. **(A)** Osteogenesis of bone marrow stem cells (BMSC) from wt and *Sirt5^-/-^* mice showed no difference as measured by alizarin red. Expression of Sirt5 mRNA and protein in RAW264.7 cells **(B)** and BMDMs **(C)** with and without osteoclast differentiation. The Sirt5 specific inhibitor MC3482 suppressed the osteoclast differentiation of RAW264.7 cells **(D)** and BMDMs **(E)**, manifested by decreased expression of osteoclast marker genes. **(F)** The Sirt5 activator resveratrol can reverse this effect of MC3482 on osteoclast differentiation in RAW264.7 cells.

**Figure S3** Lysine succinylome and SirtT5 expression during osteogenesis. **(A)**Western blot analysis of lysine succinylome, malonylome, and glutarylatome during Raw264.7 osteoclast differentiation. **(B)** Identification of highly succinylated targets associated with osteoclastogenesis.

**Figure S4** Cdc42 is a succinylated target of Sirt5. **(A-C)** MS spectrum of three succinylated sites (Lys153, Lys133, Lys163).

**Figure S5** Sirt5 interacts with Cdc42 and regulates the levels and signaling pathways and level of Cdc42. **(A)**Ectopically expressed Cdc42 and Sirt5 directly interacted with each other. **(B)**Endogenous Cdc42 colocalised with endogenous Sirt5. **(C)** *Sirt5* knockdown increased Cdc42 levels in RAW264.7 cells. **(D)***Sirt5* overexpression decreased the expression Cdc42 in Raw264.7 cells. **(E)***Sirt5-/-* mice also had higher expression of Cdc42 in osteoclasts compared to wt mice, and the Sirt5 activator resveratrol decreased the Cdc42 levels in osteoclasts from wt mice, but had no effect on preosteoclasts from *Sirt5-/-* mice. **(F)** The effect of resveratrol on Cdc42 was counteracted by the Sirt5 inhibitor MC3482.

**Figure S6** Identification of functional succinylated sites of Cdc42. (**A**) Three succinylated sites of Cdc42 overlap or are adjacent to ubiquitous sites. (**B**) Lys153 was highly conserved across species and human isoforms. K to R mutation mimicking desuccinylation for identification of functional sites.
