# Supplementary material for: Sirt5 desuccinylates Cdc42 to mediate osteoclastogenesis and bone remodeling in mice
Source: Genes Dis. 2023 Jul 3;11(3):101002. doi: 10.1016/j.gendis.2023.04.033 (PMC10806281; doi:10.1016/j.gendis.2023.04.033)
Supplement: Multimedia component 3 [file mmc3.docx]

**Materials and methods**

**Antibodies and Reagents**

Antibodies and Reagents

Commercial available antibodies were purchased from the following suppliers: Antibodies to Sirt5 (#ab259967), Cdc42 (#ab187643, #ab41429), osteopontin (Opn) (#ab283656), p-Erk1/2 (#ab201015), Erk1/2 (#ab17942), pAkt (#ab192623), Akt (#ab32505), p-p38 (#ab4822) and p38 (#ab170099) (Abcam, Cambridge, UK), Sirt5(sc-271635) (Santa Cruz Biotechnology, Heidelberg, Germany), others were antibodies against HA-tag (#bs-0966R), Flag-tag (#bsm-33346M), β-actin (#bsm-33036M) (BIOSS, Beijing, China), anti-succinyllysine mouse mAb (#PTM-419), anti-malonyllysine mouse mAb (#PTM-902), anti-glutaryllysine mouse mAb (#PTM-1152) and anti-ubiquitin rabbit mAb (#PTM-1106RM) (PTM-Biolabs, Hangzhou, China). Acid phosphatase, leukocyte (TRAP) kit (#387A) was purchased from Sigma Aldrich (St. Louis, MO, USA). Recombinant M-CSF protein (#416-ML) and RANKL (#462-TEC) were purchased from R&D Systems (Minnesota, USA).

**Mice**

Sirt5-/- C57BL/6 mice were generated by Cyagen Biosciences (Guangzhou,China) using the CRISPR/CAS9 strategy, resulting in a 427 bp deletion in the sequence of the Sirt5 gene. Mice were then randomly divided into groups. The mice were housed in cages (up to 5 mice/cage) and fed with the same sterile mouse chow (KA, Beijing, China) and sterile water and subjected to light/dark cycles at 24°C for 12 hours in a specific pathogen-free facility (SPF). Experiments were performed after at least one week of acclimation. No deaths occurred in the experimental or control groups during or after anaesthesia. All animal experiments and procedures were approved by the Ethics Committee of the First Affiliated Hospital of Shandong First Medical University.

**Cell culture and isolation**

HEK293T and pre-osteoclast RAW264.7 cell lines were obtained from the Cell Bank of Type Culture Collection of the Chinese Academy of Sciences (Shanghai, China) and were cultured in high glucose Dulbecco's modified Eagle's medium (DMEM, Gibco, Paisley, UK) containing 10% FBS, 1% penicillin/streptomycin/amphamycin mixture. Bone marrow-derived macrophages (BMDM) and bone marrow-derived mesenchymal stem cells (BM-MSC) from mouse tibia and femur were prepared and cultured as previously described.

**In vitro and ex vivo osteoblast and osteoclast differentiation**

For osteoclast differentiation, RAW264.7 cells were cultured in high glucose DMEM (Gibco, Paisley, UK) with RANKL (50 ng/mL) for 5 days, and BMDMs were cultured in DMEM with M-CSF (50ng/mL) and RANKL (50ng/mL). For osteoblast differentiation, BM-MSCs were cultured in α-MEM with 10 mmol/L, β-glycerophosphate, 50 μg/mL ascorbic acid and 200 ng/mL BMP-2. Alizarin Red S solution (#G1452, Solarbio, Beijing, China) was used for staining to assess the degree of mineralisation.

**Generation of RNA interference, site-directed mutagenesis and**

**overexpressing**

RNA interference generation, site-directed mutagenesis and overexpression Small interfering RNA specific for Sirt5 was synthesised by RIBOBIO (Ribobio, Guangzhou, China). For RNA interference, cells were cultured in complete medium in plates for 24 h and then transfected with siRNA (100 nM) using HiPerFect Transfection Reagent (Qiagen, Valencia, CA, USA) according to the manufacturer's protocol. After 48 hours, cells were harvested for further assays. A site-directed mutagenesis system (Mut Express MultiS Fast Mutagenesis Kit, Vazyme, Nanjing, China) was used to construct the mutation plasmids of Cdc42 at Lys133, Lys153 and Lys163. Transfection of the site mutation plasmids was performed using Lipofectamine 2000 Reagent (Thermo Fisher Scientific, Waltham, MA, USA). The interaction between Sirt5 and Cdc42 was confirmed by co-transfection of mouse HA-tagged Sirt5 (pcDNA3.1HA-Sirt5) and FLAG-tagged Cdc42 (pcDNA3.1FLAG-Cdc42) into HEK-293T cells. We used a GFP-fused expression vector to construct a Sirt5 overexpression plasmid and packaged lentiviruses to stably transfect RAW264.7 cells.

**Bone analyses**

To assess bone volume and static histomorphometry, mouse tibiae were scanned using micro-CT (Quantum GX2; PerkinElmer, Hopkinton, MA, USA), the projection image was reconstructed into a 3D visualisation image, and trabecular bone parameters were calculated using Caliber Analyzer software (PerkinElmer, Hopkinton, MA, USA). New bone formation was assessed by double fluorescence labelling with calcein and tetracycline. Histomorphometric analyses were performed using TRAP staining of osteoclasts and osteopontin immunostaining of osteoblasts. Serum bone remodelling markers fTRAP, BALP Elisa Kit (#D721140, #D721049) and Blood Calcium Concentration Assay Kit (#D799342) were purchased from Sangon Biotech (Shanghai, China).

**Lysine succinylome analysis**

Protein extraction from undifferentiated and differentiated RAW264.7 cells was performed with using 8 M urea with 1% protease inhibitor cocktail (Calbiochem, Billerica, MA, USA), followed by chemical digestion with 10 mM dithiothreitol followed by alkylation with 11 mM iodoacetamide. After two rounds of trypsin hydrolysis, the resulting peptides were subjected to high pH reversed-phase HPLC system. The tryptic peptides were dissolved in NETN buffer (100 mM NaCl, 1 mM EDTA, 50 mM Tris-HCl, 0.5% NP-40, pH 8.0) and incubated with Pan anti-succinyllysine antibody beads to enrich for succinylated peptides. succinylated peptides. After washing and elution, the resulting peptides were desalted with desalting using C18 Zip Tips for LC-MS/MS analysis on the Q ExactiveTM Plus and coupled online to the EASY-nLC 1000 UPLC system (Thermo Scientific, San Jose, CA, USA). The resulting MS/MS data were searched using the Maxquant search engine (v1.6.5.0). Quantitative proteomics data were used to normalise the lysine succinylome data to remove the effect of protein expression on the succinylation levels.

**Western blotting and immunoprecipitation**

For Western blotting, cells subjected to different treatments or from mice were lysed in in RIPA lysis buffer containing a protease and phosphatase inhibitor cocktail (Beyotime Biotechnology, China). A polyvinylidene difluoride membrane (Millipore; Bedford, MA, USA) was incubated with the indicated primary antibodies and an HRP-conjugated antibody and an HRP-conjugated secondary antibody. Protein bands of the cell lysates were developed using enhanced chemiluminescence and detected using the Fusion Solo2 system (Vilber Lourmat, France). For immunoprecipitation, cell extracts were lysed in IP lysis buffer (#87788, Thermo Fisher Scientific, MA, USA), primary antibodies were stirred overnight at 4°C and protein was collected using Magic Beads (#88802, Thermo Fisher Scientific, Massachusetts, USA).

**Immunofluorescence**

RAW264.7 cells were fixed in 4% paraformaldehyde and permeabilised with 0.1% Triton X-100. For staining, cells were incubated with primary antibodies overnight at 4°C and with appropriate secondary antibodies for 1 hour at room temperature and then imaged using an FV3000 confocal microscope (Olympus Corporation, Tokyo, Japan).

**Quantitative Real-Time PCR Analysis**

RNA was reverse transcribed into cDNA using HiScript reverse transcriptase (Vazyme, Nanjing, China) and analysed by SYBR Green-based quantitative RT-PCR in triplicate on the LightCycler 480 system (Roche, South San Francisco, CA, United States). Primers for PCR amplification were as follows:

| *Cdc42* | Forward primer: 5’-CCCATCGGAATATGTACCAACTG-3’ |
| --- | --- |
|  | Reverse primer: 5’-CCAAGAGTGTATGGCTCTCCAC-3’ |
| *Sirt5* | Forward primer: 5’-CTCCGGGCCGATTCATTTCC-3’ |
|  | Reverse primer: 5’-GCGTTCGCAAAACACTTCCG-3’ |
| *Mmp9* | Forward primer: 5’-GCGTCGTGATCCCCACTTAC-3’ |
|  | Reverse primer: 5’-CAGGCCGAATAGGAGCGTC-3’ |
| *Trap* | Forward primer: 5’-GCAACATCCCCTGGTATGTG-3’ |
|  | Reverse primer: 5’-GCAAACGGTAGTAAGGGCTG-3’ |
| *Rank* | Forward primer: 5’-CATCTTCGGCGTTTACTACAGG-3’ |
|  | Reverse primer: 5’-TCCACTTAGACTACTGCAAGCA-3’ |
| *Ctsk* | Forward primer: 5’-GTTACTCCAGTCAAGAACCAGG-3’ |
|  | Reverse primer: 5’-TCTGCTGCACGTATTGGAAGG-3’ |
| *Actb* | Forward primer: 5’-GTGACGTTGACATCCGTAAAGA-3’ |
|  | Reverse primer: 5’-GCCGGACTCATCGTACTCC-3’ |

**Statistics**

GraphPad Prism software (v8.2.1; GraphPad, San Diego, CA, USA) was used for statistical analysis. Data are expressed as mean ± SD. Differences between groups were determined by unpaired two-tailed Student's t-test.
